# Multiple regression analysis of a comprehensive transcriptomic data assembly elucidates mechanically- and biochemically-driven responses to focused ultrasound blood-brain barrier disruption

**DOI:** 10.7150/thno.65064

**Published:** 2021-10-11

**Authors:** Alexander S. Mathew, Catherine M. Gorick, Richard J. Price

**Affiliations:** 1 Department of Biomedical Engineering, University of Virginia, Charlottesville, VA.; 2 Department of Radiology & Medical Imaging, University of Virginia, Charlottesville, VA.

**Keywords:** focused ultrasound, blood-brain barrier, drug delivery, transcriptomics, neurogenesis

## Abstract

**Background:** Focused ultrasound (FUS) blood brain barrier disruption (BBBD) permits the noninvasive, targeted, and repeatable delivery of drugs to the brain. FUS BBBD also elicits secondary responses capable of augmenting immunotherapies, clearing amyloid-β and hyperphosphorylated tau, and driving neurogenesis. Leveraging these secondary effects will benefit from an understanding of how they correlate to the magnitude of FUS BBBD and are differentially affected by the mechanical and biochemical stimuli imparted during FUS BBBD.

**Methods:** We aggregated 75 murine transcriptomes in a multiple regression framework to identify genes expressed in proportion to biochemical (i.e. contrast MR image enhancement (CE)) or mechanical (i.e. harmonic acoustic emissions from MB-activation (MBA)) stimuli associated with FUS BBBD. Models were constructed to control for potential confounders, such as sex, anesthesia, and sequencing batch.

**Results:** MBA and CE differentially predicted expression of 1,124 genes 6 h or 24 h later. While there existed overlap in the transcripts correlated with MBA vs CE, MBA was principally predictive of expression of genes associated with endothelial reactivity while CE chiefly predicted sterile inflammation gene sets. Over-representation analysis identified transcripts not previously linked to BBBD, including actin filament organization, which is likely important for BBB recovery. Transcripts and pathways associated with neurogenesis, microglial activation, and amyloid-β clearance were significantly correlated to BBBD metrics.

**Conclusions:** The secondary effects of BBBD may have the potential to be tuned by modulating FUS parameters during BBBD, and MBA and CE may serve as independent predictors of transcriptional reactions in the brain.

## Introduction

The selection of therapeutic agents capable of accessing the central nervous system (CNS) via the vasculature is severely limited by the blood brain barrier (BBB) 1. Microbubble (MB) activation with focused ultrasound (FUS) causes temporary BBB disruption (BBBD) and represents a promising method for significantly expanding the neuropharmacological arsenal 2-4. In this regimen, MB are first administered systemically. Focused acoustic waves generated outside the skull are then applied to a region of interest, usually under MRI-guidance, causing the circulating MBs to oscillate. These oscillations impart mechanical forces on the BBB, transiently increasing its permeability. FUS-mediated BBBD is an attractive modality compared to surgery or global chemical BBBD methods, as it is non-invasive, targeted, and easily repeatable. This approach has enabled successful delivery of many therapeutics to the brain normally barred by the BBB, such as antibodies 5-7, genes 8-10, and neural stem cells 11,12. Moreover, the safety of FUS-mediated BBBD has been asserted in several studies 3,13,14 and continues to be tested in multiple clinical trials (NCT02986932, NCT03739905, NCT03119961, NCT03671889, NCT04118764).

More recently, there has been an emerging interest in so-called “secondary” effects of FUS BBBD on brain physiology 15. Depending on the FUS parameters employed, these may include acute changes in cerebral blood flow 16,17, as well as chronic responses associated with transcriptional-level changes, such as sterile inflammation (SI) 18-23, the clearance of amyloid-β 20,24 and hyperphosphorylated tau 25,26, and neurogenesis 27-30. While it is enticing to propose that these chronic secondary responses to FUS BBBD could be leveraged therapeutically, our mechanistic understanding of how FUS BBBD drives transcriptional responses in the brain is still emerging, and opportunities for significantly advancing our understanding exist. For example, despite their importance, relationships between FUS BBBD and sterile inflammation (SI) have been difficult to clearly define due to differences in experimental variables across studies, including MB dose, ultrasound frequency, and peak-negative pressure (PNP). Thus, an approach capable of universally defining relationships between FUS BBBD and SI over a spectrum of studies using unifying metrics of BBBD would be of considerable value, especially if it could identify and predict novel transcriptional pathways activated by FUS BBBD. Furthermore, during FUS BBBD, the brain is exposed to both mechanical (MB oscillation in blood vessels) and biochemical (plasma in the parenchymal space) stimuli. Understanding which, if any, transcriptional programs are differentially activated by mechanical vs. biochemical signaling could yield new insights into how FUS parameters may be tuned to better augment therapeutic bioeffects.

To this end, we deployed a “data-driven” approach, wherein transcriptome-wide multiple regression analyses were applied to a comprehensive data set comprised of 75 murine samples. Gene expression was regressed against unifying BBBD metrics associated with biochemical [i.e. MR contrast enhancement (CE)] and/or mechanical [i.e. harmonic acoustic emissions related to MB activation (MBA)] stimuli in a negative binomial framework, controlling for known confounders including sex, anesthesia, and sequencing batch. From this analysis, we identified unique genes and signaling pathways whose expression levels at 6 h and 24 h after FUS BBBD covary with the magnitude of MBA and/or CE at the time of treatment, establishing generalizable FUS-responsive transcriptional programs.

## Materials and Methods

Murine transcriptomes were acquired and processed as previously described 22,23. All transcriptomes are available through the Gene Expression Omnibus functional genomics data repository (GSE 184751). Modifications or additions to previously processed data are described below.

### Passive Cavitation Detection

We defined a metric of harmonic acoustic emissions that could be applied across multiple previous experiments in our lab. To this end, acoustic emissions data were re-analyzed using an in-house MATLAB (MathWorks) program. For each FUS-treated mouse, a fast Fourier transform (FFT) was applied to appended waveforms collected from a 2.5 mm wideband unfocused hydrophone during each FUS pulse. MBA was then defined as the ratio of the average amplitude of the top 5 peaks in a 200 Hz band surrounding the second harmonic (2.22 MHz) to the average amplitude of the top 5 peaks in 200 Hz band in a broadband region in which our hydrophone is not sensitive. The second harmonic was chosen for this metric because the hydrophone exhibits better sensitivity in this range.

### Data Aggregation and Multiple Regression

Raw RNA-seq data were generated and summarized to gene-level abundance estimates as previously described 22,23. In total, our starting dataset consisted of 75 transcriptomes spanning various experimental conditions (**Table 1**), each of which was comprised of median-of-ratios-normalized expression levels for 34,099 genes. All subsequent analyses were performed in R v4.0.0. Transcriptomes from different FUS BBBD studies were aggregated with paired CE and MBA analyses. Samples which were not FUS-treated were assigned CE and MBA values of 1. For each gene in our dataset, eight multiple regression models were then generated using DESeq2 v1.3.1 31. Briefly, DESeq2 permits construction of generalized linear models (GLMs) within a negative binomial framework, which appropriately accounts for the variability inherent to RNA-seq counts data. This enables statistical testing of the effect size of an individual experimental variable (such as a unit change in CE) on a particular gene's expression, while controlling for the effect sizes of others (such as anesthesia, sex, and sequencing batch). Each of the 8 models we constructed in this framework for each gene was a unique permutation of correlation type (linear vs exponential), continuous BBBD metric (MBA vs CE), and time point (6 h vs 24 h). Sex, anesthesia type, and sequencing batch were including as categorical covariates for each model permitting enough samples were available. Significantly correlated genes were defined as those with Benjamini-Hochberg adjusted p values less than 0.05 when testing for the effect of CE or MBA. Significant genes from pairs of linear and exponential models were merged via union. Over representation analysis (ORA) was performed for positively and negatively correlated genes from each pool using clusterProfiler v3.18.1 32 with the Gene Ontology: Biological Processes gene sets 33,34. Briefly, the Gene Ontology: Biological Processes gene sets are expert-curated collections of genes known to be associated with particular biological functions. The “Astrocyte Cell Migration” gene set, for example, contains 9 genes, *Apcdd1, Arhgef7, Ccl2, Ccl3, Ccl12, Ccr2, Gpr183, Hexb,* and* Scrib*. ORA compares a user-defined list of genes (such as those significantly correlated with CE or MBA) with each Biological Process gene set, testing whether the extent of overlap is more than would be expected by chance alone. Gene concept networks were generated using clusterProfiler. For visualization of top functional enrichments, redundant pathways were removed via semantic similarity analysis. 4-group intersections were visualized with UpSetR v.1.4.0 35.

## Results

### Data processing pipeline

**Figure 1:**
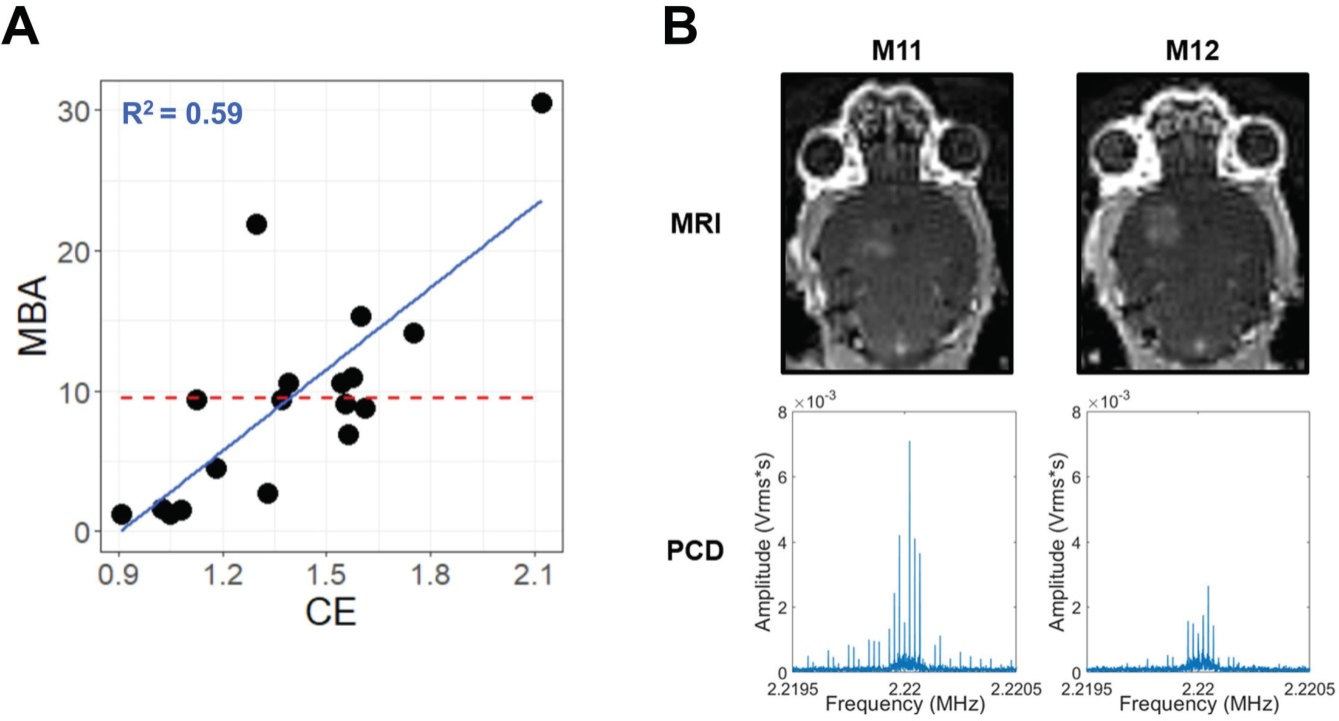
** MBA and CE are not collinear. (A)** Scatter-plot for all samples with paired CE and MBA data. The solid blue line and text represent linear regression, while the dashed red line represents the MBA mean, simulating a null linear fit. **(B)** Paired T1-weighted contrast-enhanced 3T MRI images (top) and PCD traces in the Fourier domain around the 2^nd^ harmonic (bottom) for 2 different mice (M11 and M12) during FUS BBBD treatment within a single experiment. Comparison of M11 and M12 illustrates that the relative magnitudes of MBA and CE can vary markedly from treatment to treatment.

Across multiple blood-brain barrier opening and gene delivery experiments 22,23, paired MBA and CE data had been previously acquired (**Table 1**). Analyzing these data via a linear regression of MBA against CE revealed an R^2^ value of 0.59 (**Figure 1A**). This corresponds to a Variance Inflation Factor of 2.4, indicating very low collinearity between MBA and CE. Indeed, some FUS-treated samples with high MBA had low CE, and vice versa (**Figure 1B**). Thus, we reasoned that a multiple regression analysis could permit the identification of transcripts and gene sets uniquely correlated to either MBA or CE.

**Figure 2:**
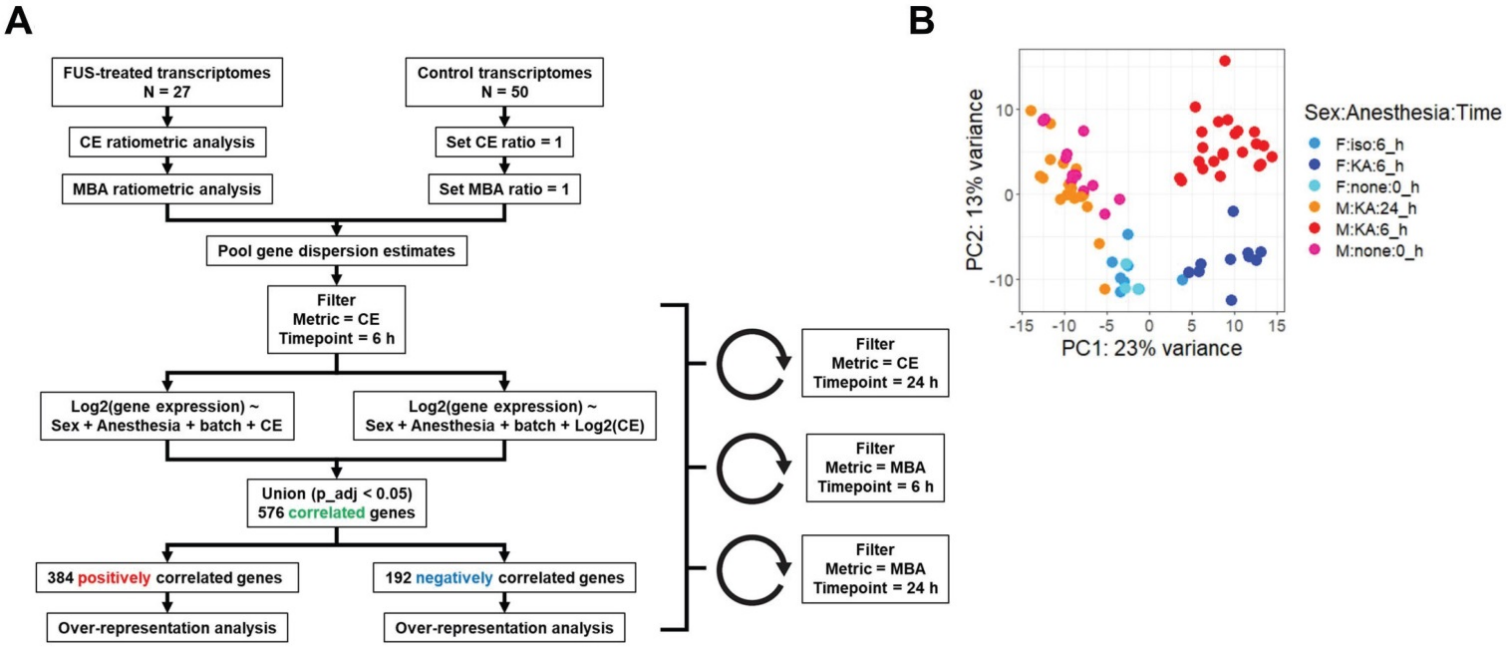
** Overview of dataset processing and variability. (A)** Flow chart describing computational processing pipeline. Untreated and FUS treated samples from multiple studies were pooled and analyzed for contrast enhancement (CE) and microbubble activation (MBA). Linear and exponential models were fit for each prediction metric (CE or MBA) and timepoint (6 h vs 24 h post treatment), followed by bioinformatics analyses. **(B)** Principle components analysis of RNA-seq transcript counts after variance stabilizing transformation. Each dot represents a single sample, color coded according to the sample characteristics including sex, anesthetic, and harvest timepoint.

We next established a multiple regression pipeline to test whether contrast enhancement (CE) or microbubble activation (MBA) could predict gene expression 6 or 24 h after treatment (**Figure 2A**). Pooling previously published studies (GSE141728, GSE152171), our dataset contained 27 transcriptomes from FUS-treated mice with paired CE analyses, 18 of which also permitted analysis of MBA. Additionally, our dataset contained 50 transcriptomes obtained from FUS-negative male and female mice treated with distinct combinations of anesthesia and microbubbles (MB). Principle components analysis (PCA) of all 75 transcriptomes revealed recent treatment with ketamine + α_2_ agonist (KA) followed by sex as the primary drivers of transcriptome-wide variability (**Figure 2B**).

Gene expression at 6 or 24 h was modeled as linear or exponential functions of CE or MBA, controlling for anesthesia, sex, and sequencing batch where possible, for a total of 8 models. Presence of MB was not included as a model variable as we have shown it has negligible effects on the murine transcriptome 22. Similarity between each linear and exponential model varied, with as much as 88% overlap in the case of the genes expressed 24 h post-treatment significantly (p adjusted < 0.05) linearly or exponentially correlated with CE, and as little as 45% for 6 h gene expression correlates of MBA (**Figure S1**). We combined significant expression correlates from each pair of models for a total of 4 pools of genes (6 h genes predicted by CE, 6 h genes predicted by MBA, 24 h genes predicted by CE, and 24 h genes predicted by MBA).

### Top Transcripts Predicted by CE and MBA after FUS BBBD

**Figure 3:**
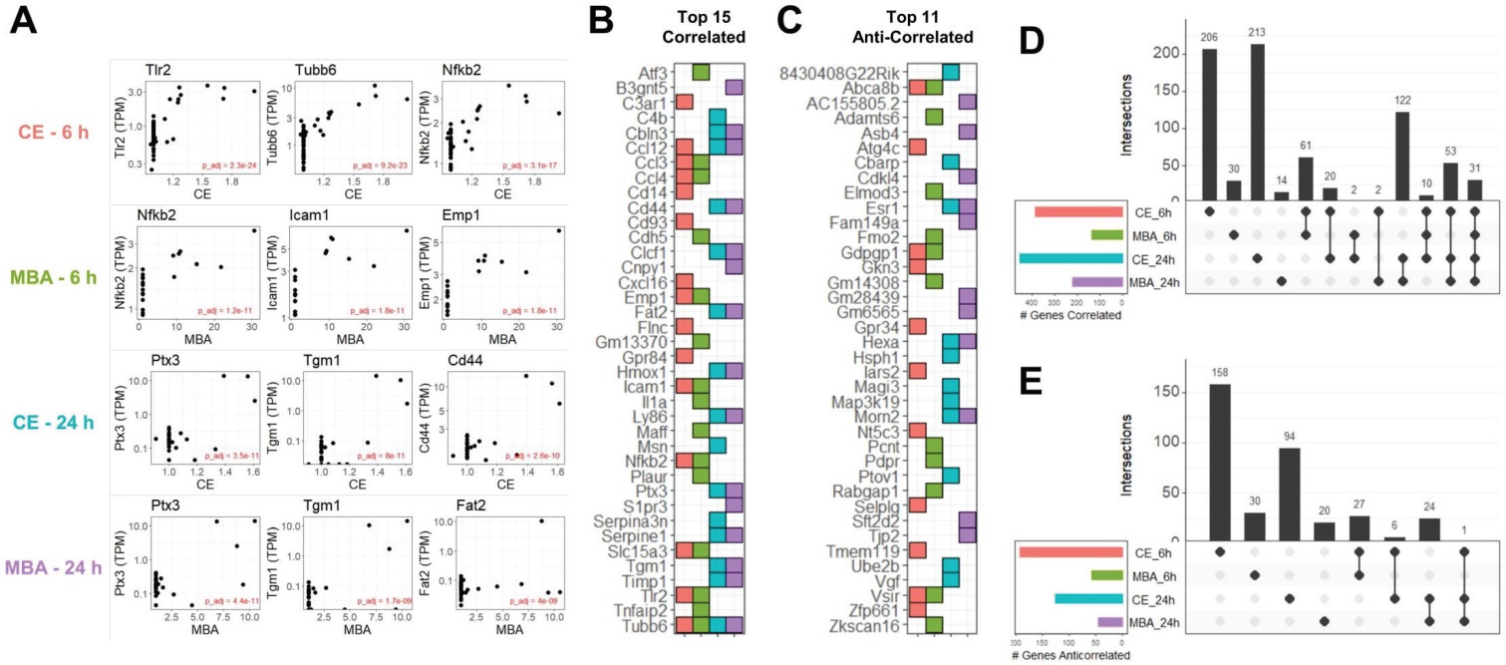
** CE and MBA predict significant gene expression 6 h and 24 h after FUS BBBD. (A)** Scatter-plots of TPM normalized expression for the top 3 genes predicted by CE or MBA at 6 h or 24 h after treatment. **(B)** Tile chart representing the top 15 genes predicted in each pool. Note that the absence of a tile for a particular pool-gene combination does not necessarily mean the gene is not significantly correlated, just that it is not in the top 15. **(C)** Tile chart representing the top 11 anti-correlated genes from each pool, with the same conditions as in B. **(D)** Upset plot indicating gene identity overlaps of positively correlated genes from each pool. **(E)** Upset plot indicate gene identity overlaps of anti-correlated genes from each pool.

In total, we identified 1,124 unique transcripts whose expression could be predicted at 6 or 24 h using CE or MBA. Multiple regression utilizing CE or MBA as continuous independent variables predicted gene expression with more sensitivity than the categorical contrast of FUS-positive vs FUS-negative (**Figure S2**). The 3 most significant genes from each pool were *Tlr2, Tubb6*, and *Nfkb2* correlated with CE at 6 h, *Nfkb2*, *Icam1,* and *Emp1* correlated with MBA at 6 h, *Ptx3*, *Tgm*, and *Cd44* correlated with CE at 24 h, and *Ptx3*, *Tgm*, and *Fat2* correlated with MBA at 24 h (**Figure 3A**). The top 15 positively correlated genes from each pool were similar, with many inflammatory transcripts such as *Icam1*, *Ccl12*, and *Ccl3*, present in at least 2 pools (**Figure 3B**). Of the 764 transcripts positively correlated with CE or MBA at 6 or 24 h, 463 were unique to a particular pool and 31 were common to all 4 (**Figure 3D**). Anti-correlated genes were less abundant but more distinct, with 302/360 transcripts unique to a particular pool and 0 transcripts common to all 4 (**Figure 3C and E**).

### Transcripts and Pathways associated with BBB Function and Repair

**Figure 4:**
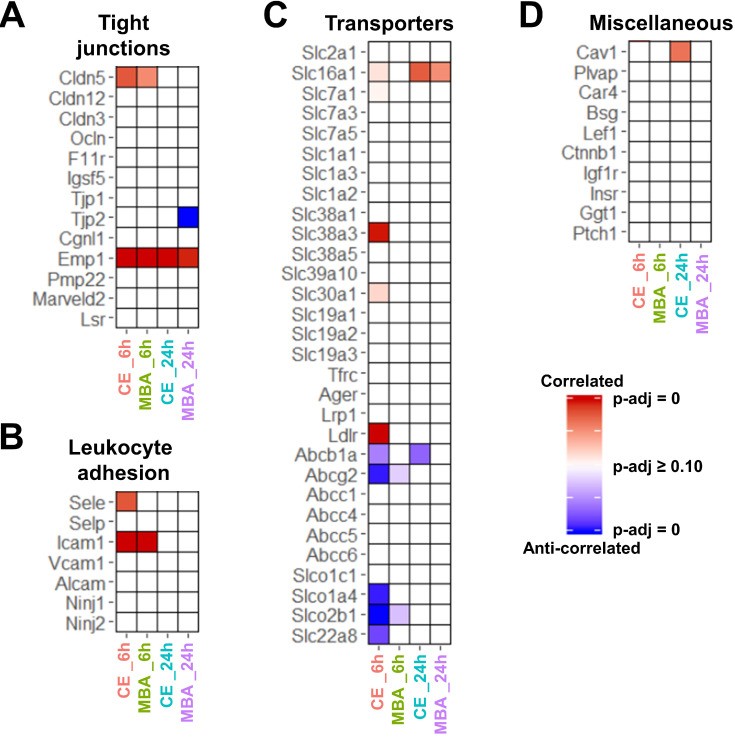
** CE and MBA predict expression of BBB associated transcripts. (A-D)** Heatmaps of significance of correlation (red) or anti-correlation (blue) for selected BBB-associated genes predicted by CE or MBA at 6 or 24 h post-FUS (columns). Selected categories include (A) tight junctions, (B) leukocyte adhesion, (C) transporters, and (D) transcytosis/miscellaneous.

**Figure 5:**
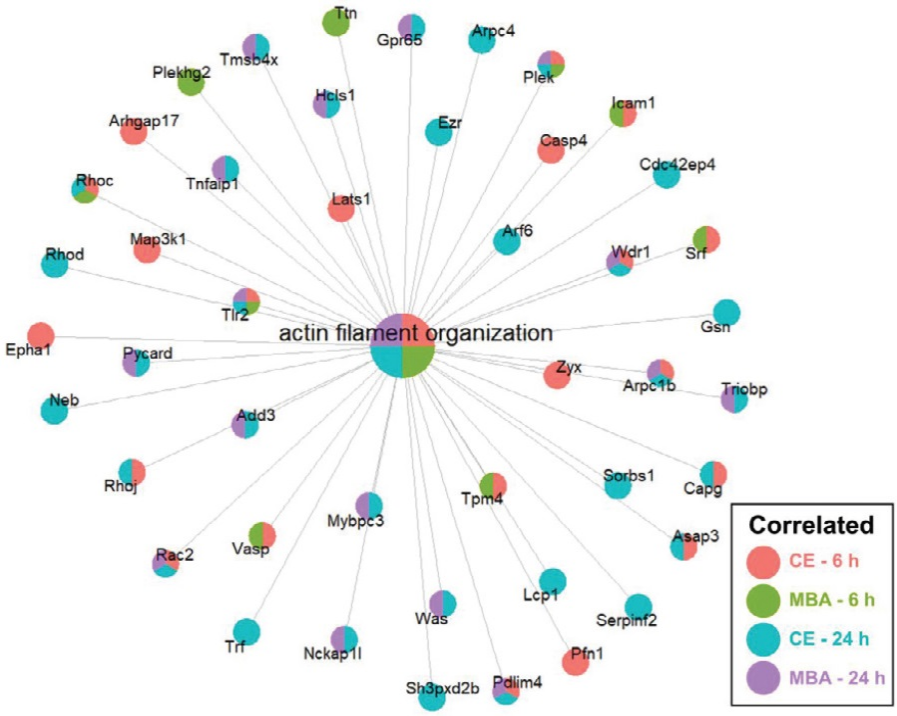
** CE and MBA robustly predict enrichment of genes associated with actin filament organization.** Integrated gene concept network for the actin filament organization pathway, which was significantly enriched across all 4 pools. Each dot, representing a contributing gene, is color coded as a pie-chart representing pools in which that gene is significantly correlated.

Next, we focused on whether CE or MBA could predict temporal expression of transcripts of interest related to BBB integrity, BBB function, and leukocyte adhesion. Among BBB tight junction transcripts, expression of *Emp1* was found to be correlated with both CE and MBA at both 6 and 24 h post-FUS (**Figure 4A**), while *Cldn5* was also significantly correlated with both metrics 6 h post-FUS. Interestingly, *Tjp2* expression 24 h after BBBD was anti-correlated with MBA. We then interrogated the expression of leukocyte adhesion molecules (**Figure 4B**). *Icam1* expression 6 h post-FUS was positively correlated with both CE and MBA. *Sele* expression was also significantly correlated with CE 6 h post-FUS. CE and MBA predicted divergent effects on expression of BBB transporters (**Figure 4C**). At 6 h post-BBBD, CE was positively correlated with expression of *Slc16a1, Slc7a1, Slc38a3, Slc30a1,* and *Ldlr*. Meanwhile, *Abcb1a, Abcg2, Slco1a4, Slco2b1, and Slc22a8* were anti-correlated with CE 6 h post-FUS. Finally, we found expression of *Cav1* 24 h post-FUS was uniquely proportional to CE (**Figure 4D**). To then test whether positively and negatively correlated BBBD gene sets from each pool were associated with broader biological processes, we performed over-representation analysis (ORA) using the Gene Ontology Biological Processes domain. Examination of all significantly enriched pathways (p adjusted < 0.01) revealed consistent CE and MBA dependent enrichment of several transcriptional programs. From this collection of enriched gene sets, “actin filament organization” was robustly predicted by all 4 pools (i.e. CE and MBA at both 6h and 24h) (**Figure 5**). Given the importance of cytoskeletal organization in defining BBB structure, this is consistent with the hypothesis that the magnitude of BBBD correlates to the activation of subsequent processes for BBB repair.

### Additional Transcriptional Pathways Activated by FUS BBBD

**Figure 6:**
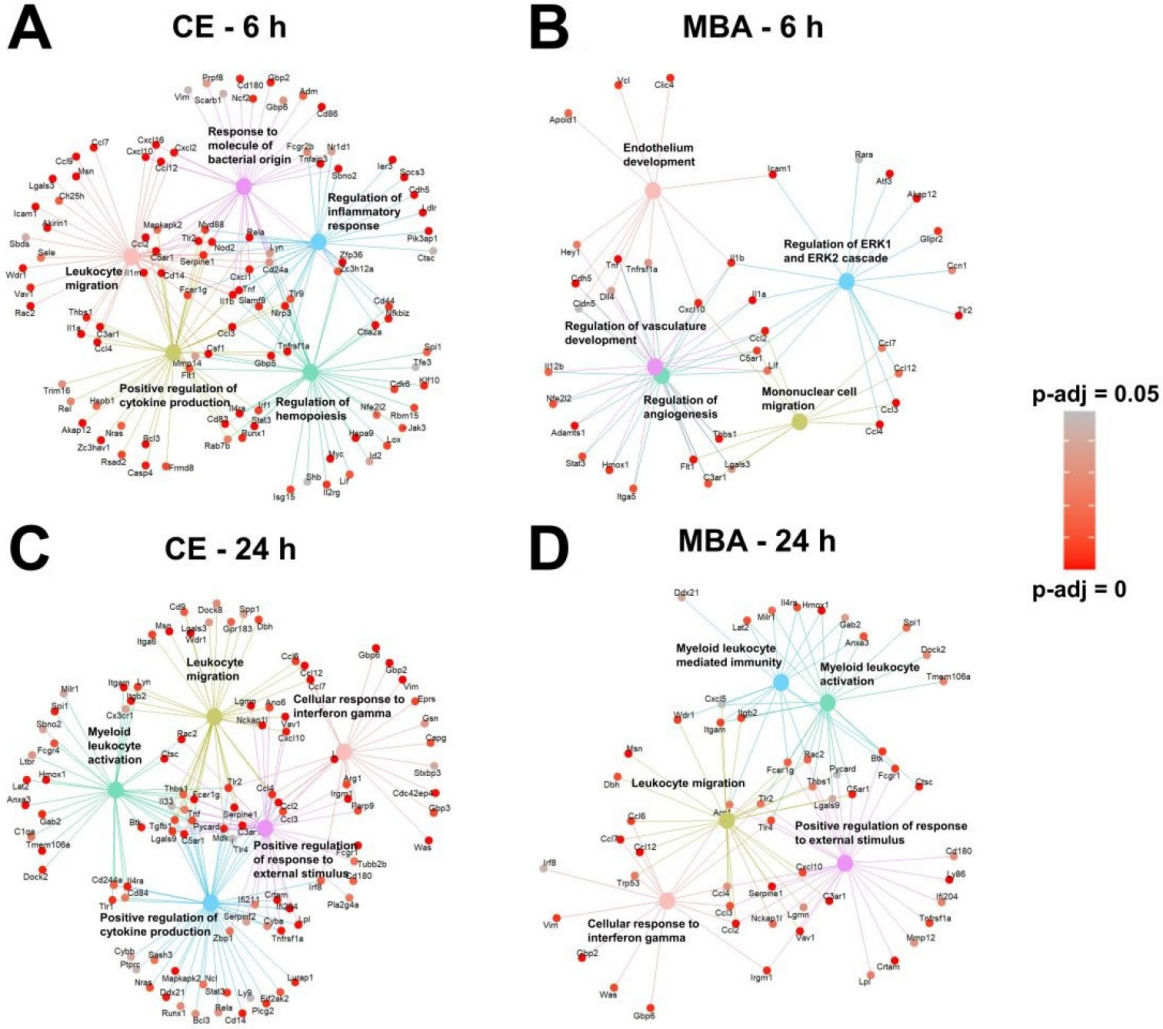
** Over-representation analysis reveals gene-sets that are most strongly associated with MBA and CE. (A-D)** Gene concept networks of the top 5 over-represented gene sets expressed 6 h after FUS proportional to CE (A), 6 h after FUS proportional to MBA (B), 24 h after FUS proportional to CE (C), and 24 h after FUS proportional to MBA (D). Redundant pathways were removed by semantic similarity analysis. Supporting genes within each network are colored in proportion to the significance of their correlation with the specified metric at the specific timepoint.

Returning to the gene set over-representation analysis, we examined the identities of the top 5 most significantly enriched pathways and their supporting transcripts for each correlate pool (**Figure 6**). As expected given the representation of transcripts associated with inflammation and immunity (**Figure 3**), all 4 pools predicted gene sets associated with leukocyte migration and/or activation. In particular, genes expressed proportional to CE 6 h post-FUS were skewed toward acute sterile inflammatory responses, such as toll-like receptor signaling (**Figure 6A**). Conversely, genes expressed 24 h post-FUS proportional to CE and MBA were more associated with subacute sterile inflammation, indicated by enriched interferon gamma signaling (**Figure 6C and D**). Interestingly, several of the top biological processes correlated with MBA 6 h post-FUS were associated with vasculature repair and development (**Figure 6B**), consistent with the concept of the MBA variable representing a mechanical stimulus concentrated on cells comprising microvessel walls (i.e. endothelium, pericytes, and smooth muscle).

**Figure 7:**
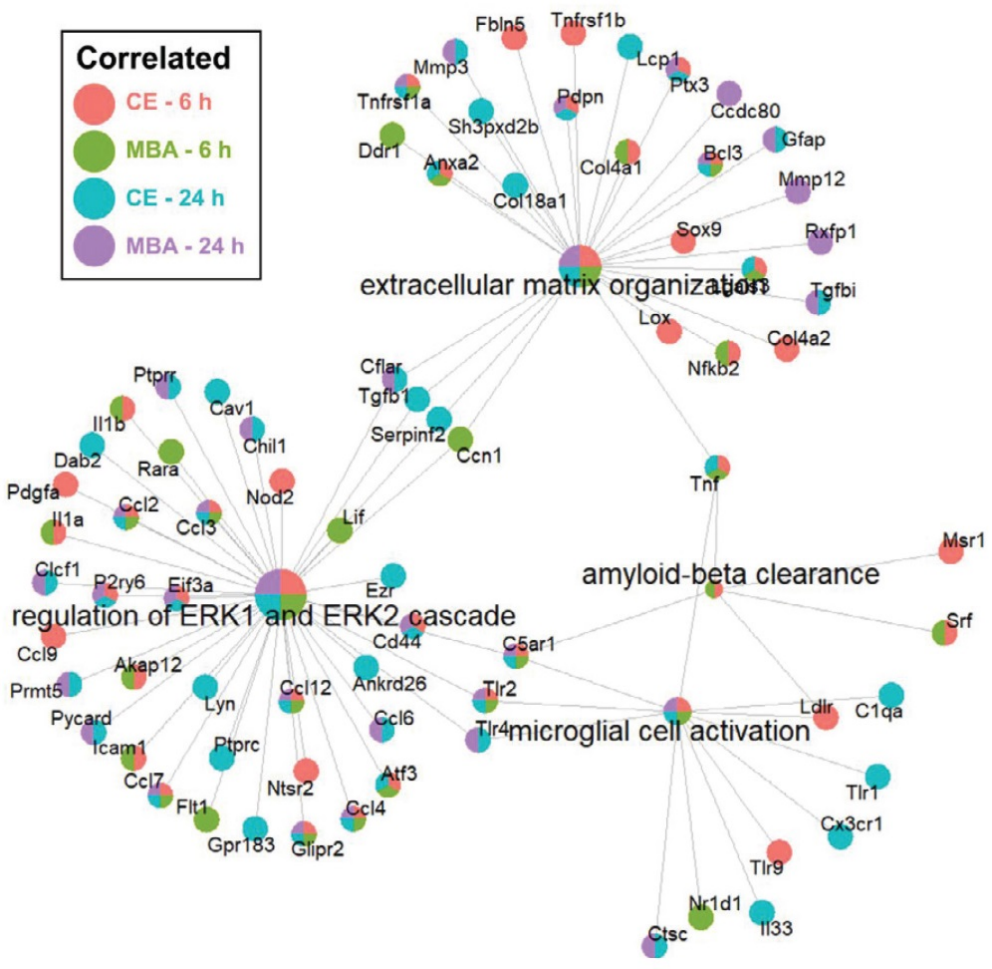
** Over-representation analysis of gene-sets associated with neurogenesis and amyloid-beta clearance.** Integrated gene concept networks are shown for the extracellular matrix organization, regulation of ERK1 and ERK2 cascade, amyloid-beta clearance, and microglial cell activation pathways. Each dot, representing a contributing gene, is color coded as a pie-chart representing pools in which that gene is significantly correlated.

Next, we sought to identify enriched pathways related to other key secondary responses of FUS BBBD identified in previous studies. We focused on neurogenesis 27-30 and amyloid-β clearance 20,24, which may be driven by microglial activation. Importantly, as shown in **Figure 7**, the “microglial activation” gene set was enriched for CE and MBA, both at 6h and 24h. Further, the “amyloid-β clearance” gene set was enriched for both CE and MBA at 6h. With regard to neurogenesis, it has been recently proposed that neurogenesis after low-intensity scanning US application and BBBD may be related to changes in ERK expression and/or perineuronal nets, a feature of the brain extracellular matrix 30. Notably, the “extracellular matrix organization” and “regulation of ERK1 and ERK2 cascade” gene sets were robustly enriched for CE and MBA, both at 6h and 24h (**Figure 7**).

### Transcripts Predicted Uniquely by Either CE or MBA after FUS BBBD

To further separate the biochemical and mechanical impacts of FUS BBBD on the transcriptome, we identified transcripts that were uniquely predicted by either CE or MBA. At 6 h post-FUS, CE predicted the expression of 280 transcripts that were not predicted by MBA, while MBA predicted 32 unique transcripts that were not predicted by CE. At 24h post-FUS, CE predicted 245 transcripts that were not predicted by MBA, while MBA predicted 16 transcripts that were not predicted by CE. **Table 2** shows the top 20 uniquely predicted transcripts for each case.

## Discussion

FUS mediated BBBD permits the targeted, non-invasive, and repeatable delivery of therapeutics from the bloodstream to the CNS, with several clinical trials now underway (NCT02986932, NCT03739905, NCT03119961, NCT03671889, NCT04118764). Recently, there has also been considerable interest in better understanding and therapeutically leveraging effects that occur secondary to FUS BBBD. These secondary effects include enhanced penetration of therapeutics (e.g. nanoparticles) through tissue 36,37, activation of neurogenesis 27,28,30, amyloid-β 20,24 and hyperphosphorylated tau clearance 26, and even sterile inflammation 18,38, which could augment immunotherapies. Nonetheless, our understanding of these consequences of FUS BBBD has been complicated by variability across experimental parameters. Moreover, while separating the mechanical impact of FUS BBBD (i.e. MB oscillation in capillaries) from the biochemical impact of FUS BBBD (i.e. exposure of brain tissue to plasma constituents) could yield insight, no existing empirical approaches definitively delineate their respective contributions. Here, to both extend our understanding of the impact of FUS BBBD on the brain transcriptome and potentially disaggregate the relative impacts of mechanical and biochemical stimuli during FUS BBBD, we employed a data driven approach that combined CE and MBA measurements with 75 separate transcriptional data sets. CE and MBA served as independent predictors of gene expression 6 h and 24 h post-treatment. By pooling datasets across experimental conditions and including these as model covariates, we extend the generalizability of our results to other experimental conditions and FUS parameters. We identified over 1000 distinct genes that are expressed 6 h or 24 h post FUS in proportion to the magnitude of CE or MBA, several of which were directly associated with BBB structure and function. Expression of a substantial number of genes was unique to a particular time point. Notably, many transcripts were also uniquely dependent on either MBA or CE, suggesting that both the mechanical and biochemical perturbations created by FUS BBBD can significantly and differentially affect transcriptional responses. Furthermore, consistent with the hypothesis that the mechanical component associated with FUS BBBD will preferentially affect the endothelium, gene sets expressed 6 h post-FUS in response to MBA specifically were most strongly associated with endothelial activity and repair. Importantly, common to all models was an enrichment for genes associated with actin filament organization, suggesting a possible new mechanism for BBB restoration. Ultimately, our results indicate that MBA and CE can independently predict transcriptional responses underlying important secondary effects to FUS BBBD.

### Validation and Power of Data-Driven Approach

A central concept of our investigation was enabled by the observation that CE and MBA are not collinear variables (Figure 1), thereby permitting delineation of their relative contributions to FUS BBBD-driven transcriptional changes. Of note, a similar analysis of the linear correlation between contrast enhancement and 2^nd^ harmonic emission returned an R value of 0.77 (R^2^ = 0.59) 39, matching the value obtained here. While CE and MBA are both used to monitor FUS BBBD treatment, they represent distinct phenomena. CE depends upon the accumulation of contrast agent in the brain parenchyma due to increased BBB permeability, and we argue it is a proxy for exposure of brain tissue to the biochemical milieu of plasma. On the other hand, MBA is a measure of the magnitude of intravascular MB oscillation in response to FUS, and we argue it is a proxy for mechanical perturbation of brain tissue, albeit with the greatest impact on BBB endothelium. The fact that roughly 40% of the variance in CE could not be explained by variance in MBA led us to hypothesize that each metric may have unique predictive value for gene expression after FUS application. Indeed, many of the transcripts whose expression was predicted by CE were not predicted by MBA, and vice versa (**Table 2**). Interestingly, unique predictions were more marked for CE, with MBA predicting expression of fewer genes overall, most of which were also predicted by CE. Thus, we postulate that biochemical stimuli (i.e. exposure of brain tissue to plasma) predominantly drive transcriptional responses to FUS BBBD.

One major advantage of the approach reported here is that it exhibits several statistical advantages compared to past studies of the transcriptional effects of FUS on the BBB. Integrating data from multiple experiments with variation in PNP, MB type, anesthesia, sex, and time point produced a large data set (75 transcriptomes). This allowed us to improve gene dispersion estimates and explicitly control for confounder variables in multiple regression models. We utilized established bioinformatics tools to construct multiple regressions in a negative binomial framework that appropriately models gene expression as a function of categorical and continuous experimental variables. Finally, our approach of leveraging the continuous nature of CE or MBA returned many fold more transcripts with higher confidence than simply testing the effect of FUS treatment as a categorical variable.

### Sterile Inflammation and FUS-Mediated Blood-Brain Barrier Disruption

Several FUS parameters influence the extent of SI responses after BBBD. In a study concluding SI is dependent on MB dose, the dosing schema leading to the most pronounced SI also elicited the strongest CE signatures, whether PNP was feedback-controlled or fixed 38. The authors noted this relationship, identifying significant correlations between 9 stress-related genes and CE using linear least-squares regression. In a study from our group utilizing cationic microbubbles for gene therapy, we report 0.4 MPa FUS elicits significant upregulation of SI cytokines relative to 0.1 MPa or 0.2 MPa FUS 23. Roughly, PNPs of 0.1, 0.2 and 0.4 MPa FUS lead to increases in CE of 0%, 25%, and 75% respectively. Finally, in a separate study of the effect of anesthesia on BBBD, we observed isoflurane (Iso) predisposes the BBB to more marked CE compared to ketamine + α_2_ agonist (KA) when PNP and MB dose were kept constant 22. Indeed, while BBBD induced signatures of SI under both anesthetics, the responses were more marked when FUS was applied under Iso. One corollary of the studies from our group was that neither albumin nor cationic lipid shelled MB affected the transcriptome in the absence of FUS. Thus, we propose that MBA and CE are powerful determinants of post-FUS gene expression.

Across all 4 models, we identified expression of 1,124 genes predicted by the magnitude of CE or MBA. Many positively correlated genes were associated with SI, such as *Nfkb2*, *Tnf, Tlr2, Ccl12, Cd14, Il1a, Il1b,* and *Ccl12*, consistent with previous studies 18,38. ORA revealed that SI pathways, such as “Leukocyte Migration”, “Regulation to molecule of bacterial origin”, and “Positive Regulation of Cytokine Production” were the most consistently and strongly enriched among genes positively correlated with CE or MBA. SI is primarily considered to be an innate immune response. Interestingly, signatures of adaptive immunity also appeared to be predicted by CE or MBA, especially at 24 h. *Cd44*, a lymphocyte surface glycoprotein that aids in adhesion to endothelial cells and commonly-used marker for T-cell activation, was one of the strongest correlates of both CE and MBA 24 h after treatment. “Cellular response to interferon-gamma” was one of the most enriched gene sets 24 h post-FUS, implicating multiple guanylate-binding proteins (GBPs), including *Gbp2, Gpb3,* and *Gbp6*. Thus, these analyses 1) demonstrate MBA and CE similarly predict SI, 2) highlight previously unreported families of pro-inflammatory transcripts induced by FUS BBBD, and 3) suggest that, at least at the transcriptional level, induced SI responses exist on a continuum, initiated by even minor perturbations of the BBB.

### Blood-Brain Barrier Function and Restoration

Interestingly, both CE and MBA predicted differential expression of genes directly involved with BBB function The positive correlations of the adhesion molecules *Icam1* and *Sele* are consistent with an inflamed BBB endothelium, in agreement with previous results 18,23,38. A mixture of positive and negative correlations of transporters with BBBD has been reported previously 40, and could reflect mechanisms to reestablish ionic and metabolic homeostasis in the brain parenchyma. Interestingly, we also observed correlations between CE and expression of *Cav1* 24 hours later. Upregulation of caveolin-1 expression after FUS BBBD mediates transcellular transport across the BBB 41,42, representing an alternative to the paracellular mechanism for how FUS enhances BBB permeability. Our data support both phenomena.

We also identified transcripts and transcriptional programs that may be involved in barrier reactivity and repair after FUS. It is well known that FUS BBBD is transient, but the mechanisms engaged during repair are still unclear, and knowledge of them could be remarkably useful. Of particular interest, we observed that epithelial membrane protein 1 (*Emp1*), which mediates the assembly of the BBB 43, was strongly correlated across all 4 models. In addition, *Apold1*, which was predicted uniquely by MBA at 6h, is an endothelial cell early response protein that may play a role in regulation of endothelial cell signaling and blood-brain barrier integrity 44,45. Among the top over-represented gene sets correlated with MBA were “Endothelium development”, “Regulation of vasculature development”, and “Regulation of angiogenesis” (Figure 6B). Constituent, non-inflammatory genes contributing to these pathways' enrichment code for proteins mediating tight junctions (*Cldn5, Cdh5*), VEGF signaling (*Flt1, Dll4, Hey1),* and basement membrane interaction (*Itga5, Adamts1, Lgals3, Vcl).* The fact that these pathways were most strongly associated with MBA specifically suggests that these may be the direct consequence of the mechanical forces MB impart on the vasculature.

An interesting family of transcripts consistently overrepresented in gene sets correlated with both CE and MBA was “Actin filament organization” (Figure 5). The actin cytoskeleton has been proposed to be a crucial mediator of BBB permeability. Actin provides anchoring support to tight junction proteins critical to the BBB such as JAM-1 46,47 and ZO-1 48-50. Additionally, temporospatial reorganization and dynamic expression alterations of actin modulate tight junction complexes, suggesting an active role of the cytoskeleton in modulating the BBB structure 51-54. Studies of CNS hypoxia, wherein BBB integrity is compromised, demonstrate redistributions of actin 55. Reoxygenation of hypoxic tissue then leads to rapid actin polymerization, thickening, and redistribution as barrier integrity is reestablished 56. Our data suggest a similar phenomenon may occur in response to FUS BBBD.

### Neurogenesis and Amyloid-β Clearance

Our results also support recent studies from other investigators suggesting that FUS BBBD may stimulate neurogenesis 27-30, as well as amyloid-β clearance through a microglia-dependent mechanism 20,24. For example, our over-representation analyses revealed enrichment of the “microglial activation” gene set for both variables at both timepoints. Further, for both CE and MBA at 6h, the “amyloid-β clearance” gene set was significantly enriched. Of significance for neurogenesis, both the “extracellular matrix organization” and “regulation of ERK1 and ERK2 cascade” gene sets were robustly enriched for CE and MBA, both at 6h and 24h. This may be significant because neurogenesis after BBBD over a relatively large volume of the brain has been proposed to occur due to changes in the extracellular matrix regulating perineuronal nets, as well as ERK expression and signaling (**Figure 7**).

We also identified significantly enriched transcripts associated with neurogenesis and amyloid-β clearance, especially within the set of transcripts uniquely expressed in response to MBA at 6h. For example, *A2m* has the potential to clear and degrade amyloid-β 57,58. Potentially related to neurogenesis, *Maff* has been identified as an NGF-responsive gene 59, suggesting a role for *Maff* in neuronal cell division and development. Notably, *Maff* has also been shown to abate epidermal growth factor (EGF)-induced MAPK signaling^60^, which plays a crucial role in neuronal differentiation. Finally, *Per1* may play a role in memory related signaling via gating of memory-relevant pathways in hippocampus 61.

## Supplementary Material

Supplementary figures.

## Figures and Tables

**Table 1:**
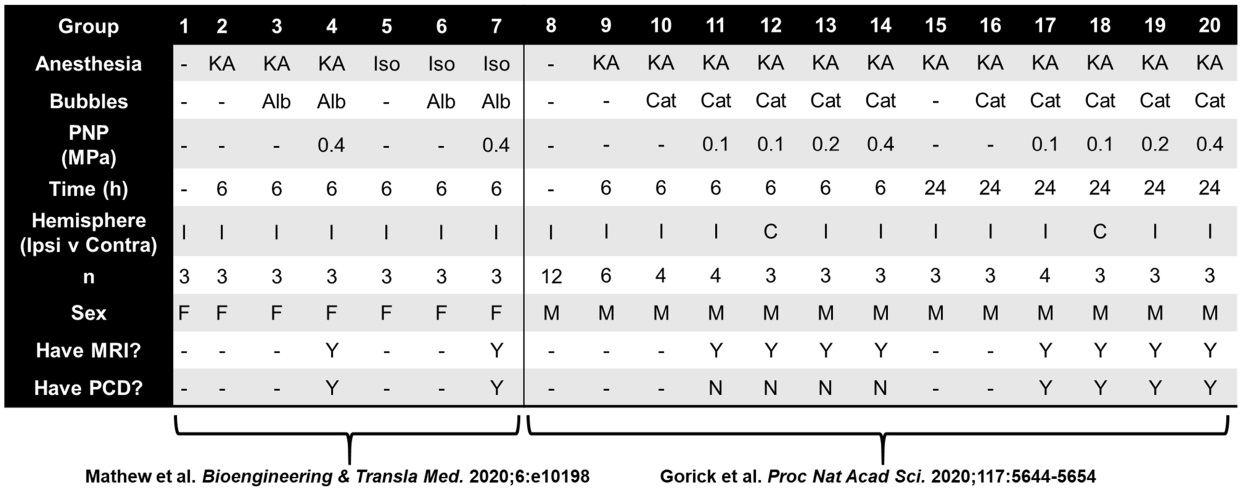
Contingency table for 75 transcriptomes used in multiple regression analyses

KA, ketamine anesthesia; Iso, isolflurane anesthesia; Alb, albumin-shelled; Cat, cationic lipid-shelled; PCD, passive cavitation detection to determine microbubble activity.

**Table 2:**
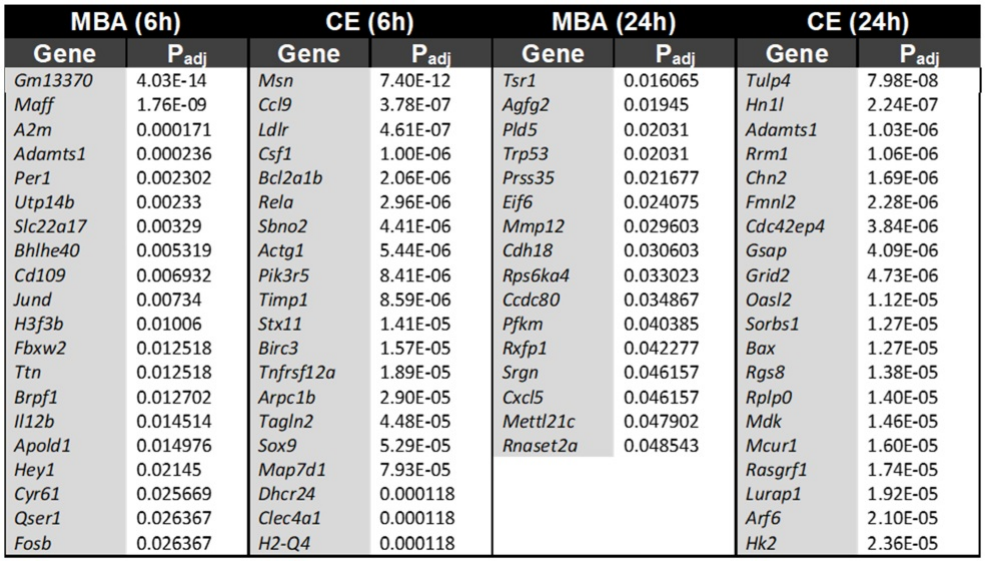
Transcripts uniquely predicted by either MBA or CE

## References

[1] Pardridge WM (2005). The blood-brain barrier: bottleneck in brain drug development. NeuroRx.

[2] Choi JJ, Pernot M, Small SA, Konofagou EE (2007). Noninvasive, transcranial and localized opening of the blood-brain barrier using focused ultrasound in mice. Ultrasound Med Biol.

[3] Hynynen K, McDannold N, Sheikov NA, Jolesz FA, Vykhodtseva N (2005). Local and reversible blood-brain barrier disruption by noninvasive focused ultrasound at frequencies suitable for trans-skull sonications. Neuroimage.

[4] Hynynen K, McDannold N, Vykhodtseva N, Jolesz FA (2001). Noninvasive MR Imaging-guided Focal Opening of the Blood-Brain Barrier in Rabbits. Radiology.

[5] Kinoshita M, McDannold N, Jolesz FA, Hynynen K (2006). Noninvasive localized delivery of Herceptin to the mouse brain by MRI-guided focused ultrasound-induced blood-brain barrier disruption. Proc Natl Acad Sci U S A.

[6] Park E-JJ, Zhang Y-ZZ, Vykhodtseva N, McDannold N (2012). Ultrasound-mediated blood-brain/blood-tumor barrier disruption improves outcomes with trastuzumab in a breast cancer brain metastasis model. J Control Release.

[7] Janowicz PW, Leinenga G, Götz J, Nisbet RM (2019). Ultrasound-mediated blood-brain barrier opening enhances delivery of therapeutically relevant formats of a tau-specific antibody. Sci Rep.

[8] Stavarache MA, Petersen N, Jurgens EM (2018). Safe and stable noninvasive focal gene delivery to the mammalian brain following focused ultrasound. J Neurosurg.

[9] Mead BP, Kim N, Miller GW (2017). Novel Focused Ultrasound Gene Therapy Approach Noninvasively Restores Dopaminergic Neuron Function in a Rat Parkinson's Disease Model. Nano Lett.

[10] Lin CJ, Lin CY, Lin YT (2019). Microbubble-facilitated ultrasound pulsation promotes direct α-synuclein gene delivery. Biochem Biophys Res Commun.

[11] Shen W-B, Anastasiadis P, Nguyen B (2017). Magnetic Enhancement of Stem Cell-Targeted Delivery into the Brain Following MR-Guided Focused Ultrasound for Opening the Blood-Brain Barrier. Cell Transplant.

[12] Burgess A, Ayala-Grosso CA, Ganguly M (2011). Targeted delivery of neural stem cells to the brain using MRI-guided focused ultrasound to disrupt the blood-brain barrier. PLoS One.

[13] Choi JJ, Selert K, Gao Z, Samiotaki G, Baseri B, Konofagou EE (2011). Noninvasive and localized blood-brain barrier disruption using focused ultrasound can be achieved at short pulse lengths and low pulse repetition frequencies. J Cereb Blood Flow Metab.

[14] McDannold N, Vykhodtseva N, Raymond S, Jolesz FA, Hynynen K (2005). MRI-guided targeted blood-brain barrier disruption with focused ultrasound: Histological findings in rabbits. Ultrasound Med Biol.

[15] Todd N, Angolano C, Ferran C, Devor A, Borsook D, McDannold N (2020). Secondary effects on brain physiology caused by focused ultrasound-mediated disruption of the blood-brain barrier. J Control Release.

[16] Todd N, Zhang Y, Livingstone M, Borsook D, McDannold N (2019). The neurovascular response is attenuated by focused ultrasound-mediated disruption of the blood-brain barrier. Neuroimage.

[17] Raymond SB, Skoch J, Hynynen K, Bacskai BJ (2007). Multiphoton imaging of ultrasound/Optison mediated cerebrovascular effects *in vivo*. J Cereb Blood Flow Metab.

[18] Kovacs ZI, Kim S, Jikaria N (2017). Disrupting the blood-brain barrier by focused ultrasound induces sterile inflammation. Proc Natl Acad Sci.

[19] Curley CT, Stevens AD, Mathew AS (2020). Immunomodulation of intracranial melanoma in response to blood-tumor barrier opening with focused ultrasound. Theranostics.

[20] Leinenga G, Götz J (2015). Scanning ultrasound removes amyloid-β and restores memory in an Alzheimer's disease mouse model. Sci Transl Med.

[21] Kovacs ZI, Burks SR, Frank JA (2018). Focused ultrasound with microbubbles induces sterile inflammatory response proportional to the blood brain barrier opening: Attention to experimental conditions. Theranostics.

[22] Mathew AS, Gorick CM, Thim EA (2020). Transcriptomic response of brain tissue to focused ultrasound-mediated blood-brain barrier disruption depends strongly on anesthesia. Bioeng Transl Med.

[23] Gorick CM, Mathew AS, Garrison WJ (2020). Sonoselective transfection of cerebral vasculature without blood-brain barrier disruption. Proc Natl Acad Sci U S A.

[24] Jordão JF, Thévenot E, Markham-Coultes K (2013). Amyloid-β plaque reduction, endogenous antibody delivery and glial activation by brain-targeted, transcranial focused ultrasound. Exp Neurol.

[25] Pandit R, Leinenga G, Götz J (2019). Repeated ultrasound treatment of tau transgenic mice clears neuronal tau by autophagy and improves behavioral functions. Theranostics.

[26] Karakatsani ME, Kugelman T, Ji R (2019). Unilateral focused ultrasound-induced blood-brain barrier opening reduces phosphorylated Tau from the rTg4510 mouse model. Theranostics.

[27] Scarcelli T, Jordão JF, O'reilly MA, Ellens N, Hynynen K, Aubert I (2014). Stimulation of hippocampal neurogenesis by transcranial focused ultrasound and microbubbles in adult mice. Brain Stimul.

[28] Mooney SJ, Shah K, Yeung S, Burgess A, Aubert I, Hynynen K (2016). Focused ultrasound-induced neurogenesis requires an increase in blood-brain barrier permeability. PLoS One.

[29] Shin J, Kong C, Lee J (2019). Focused ultrasound-induced blood-brain barrier opening improves adult hippocampal neurogenesis and cognitive function in a cholinergic degeneration dementia rat model. Alzheimer's Res Ther.

[30] Blackmore DG, Turpin F, Palliyaguru T (2021). Low-intensity ultrasound restores long-term potentiation and memory in senescent mice through pleiotropic mechanisms including NMDAR signaling. Mol Psychiatry.

[31] Love MI, Huber W, Anders S (2014). Moderated estimation of fold change and dispersion for RNA-seq data with DESeq2. Genome Biol.

[32] Yu G, Wang LG, Han Y, He QY (2012). ClusterProfiler: An R package for comparing biological themes among gene clusters. Omi A J Integr Biol.

[33] The Gene Ontology Consortium (2018). The Gene Ontology Resource: 20 years and still GOing strong. Nucleic Acids Res.

[34] Ashburner M, Ball CA, Blake JA (2000). Gene ontology: Tool for the unification of biology. Nat Genet.

[35] Conway JR, Lex A, Gehlenborg N (2017). UpSetR: an R package for the visualization of intersecting sets and their properties. Bioinformatics.

[36] Mead BP, Curley CT, Kim N (2019). Focused Ultrasound Preconditioning for Augmented Nanoparticle Penetration and Efficacy in the Central Nervous System. Small.

[37] Curley CT, Mead BP, Negron K (2020). Augmentation of Brain Tumor Interstitial Flow via Focused Ultrasound Promotes Brain-Penetrating Nanoparticle Dispersion and Transfection. Sci Adv.

[38] McMahon D, Hynynen K (2017). Acute Inflammatory Response Following Increased Blood-Brain Barrier Permeability Induced by Focused Ultrasound is Dependent on Microbubble Dose. Theranostics.

[39] McDannold N, Vykhodtseva N, Hynynen K (2006). Targeted disruption of the blood-brain barrier with focused ultrasound: association with cavitation activity. Phys Med Biol.

[40] Mcmahon D, Bendayan R, Hynynen K (2017). Acute effects of focused ultrasound-induced increases in blood-brain barrier permeability on rat microvascular transcriptome. Sci Rep.

[41] Deng J, Huang Q, Wang F (2012). The role of caveolin-1 in blood-brain barrier disruption induced by focused ultrasound combined with microbubbles. J Mol Neurosci.

[42] Pandit R, Koh WK, Sullivan RKP, Palliyaguru T, Parton RG, Götz J (2020). Role for caveolin-mediated transcytosis in facilitating transport of large cargoes into the brain via ultrasound. J Control Release.

[43] Bangsow T, Baumann E, Bangsow C (2008). The epithelial membrane protein 1 is a novel tight junction protein of the blood-brain barrier. J Cereb Blood Flow Metab.

[44] Regard JB, Scheek S, Borbiev T (2004). Verge: A Novel Vascular Early Response Gene. J Neurosci.

[45] Liu F, Turtzo LC, Li J (2012). Loss of vascular early response gene reduces edema formation after experimental stroke. Exp Transl Stroke Med.

[46] Liu Y, Nusrat A, Schnell FJ (2000). Human junction adhesion molecule regulates tight junction resealing in epithelia. J Cell Sci.

[47] Martìn-Padura I, Lostaglio S, Schneemann M (1998). Junctional adhesion molecule, a novel member of the immunoglobulin superfamily that distributes at intercellular junctions and modulates monocyte transmigration. J Cell Biol.

[48] Haskins J, Gu L, Wittchen ES, Hibbard J, Stevenson BR (1998). ZO-3, a novel member of the MAGUK protein family found at the tight junction, interacts with ZO-1 and occludin. J Cell Biol.

[49] Itoh M, Furuse M, Morita K, Kubota K, Saitou M, Tsukita S (1999). Direct binding of three tight junction-associated MAGUKs, ZO-1, ZO-2, and ZO-3, with the COOH termini of claudins. J Cell Biol.

[50] Bazzoni G, Martínez-Estrada OM, Orsenigo F, Cordenonsi M, Citi S, Dejana E (2000). Interaction of junctional adhesion molecule with the tight junction components ZO-1, cingulin, and occludin. J Biol Chem.

[51] Florian P, Schöneberg T, Schulzke JD, Fromm M, Gitter AH (2002). Single-cell epithelial defects close rapidly by an actinomyosin purse string mechanism with functional tight junctions. J Physiol.

[52] Madara JL (1987). Intestinal absorptive cell tight junctions are linked to cytoskeleton. Am J Physiol - Cell Physiol.

[53] Madara JL, Stafford J (1989). Interferon-γ directly affects barrier function of cultured intestinal epithelial monolayers. J Clin Invest.

[54] Ingber DE, Prusty D, Sun Z, Betensky H, Wang N (1995). Cell shape, cytoskeletal mechanics, and cell cycle control in angiogenesis. J Biomech.

[55] Mark KS, Davis TP (2002). Cerebral microvascular changes in permeability and tight junctions induced by hypoxia-reoxygenation. Am J Physiol - Hear Circ Physiol.

[56] Crawford LE, Milliken EE, Irani K (1996). Superoxide-mediated actin response in post-hypoxic endothelial cells. J Biol Chem.

[57] Hughes SR, Khorkova O, Goyal S (1998). α2-macroglobulin associates with β-amyloid peptide and prevents fibril formation. Proc Natl Acad Sci USA.

[58] Varma VR, Varma S, An Y (2017). Alpha-2 macroglobulin in Alzheimer's disease: A marker of neuronal injury through the RCAN1 pathway. Mol Psychiatry.

[59] Dijkmans TF, van Hooijdonk LWA, Schouten TG, Kamphorst JT, Fitzsimons CP, Vreugdenhil E (2009). Identification of new Nerve Growth Factor-responsive immediate-early genes. Brain Res.

[60] Amit I, Citri A, Shay T (2007). A module of negative feedback regulators defines growth factor signaling. Nat Genet.

[61] Rawashdeh O, Jilg A, Maronde E, Fahrenkrug J, Stehle JH (2016). Period1 gates the circadian modulation of memory-relevant signaling in mouse hippocampus by regulating the nuclear shuttling of the CREB kinase pP90RSK. J Neurochem.

