# Supplementary material for: Multiple regression analysis of a comprehensive transcriptomic data assembly elucidates mechanically- and biochemically-driven responses to focused ultrasound blood-brain barrier disruption
Source: Theranostics. 2021 Oct 11;11(20):9847–58. doi: 10.7150/thno.65064 (PMC8581408; doi:10.7150/thno.65064)
Supplement: Supplementary file 1 — Supplementary figures. [file thnov11p9847s1.pdf]

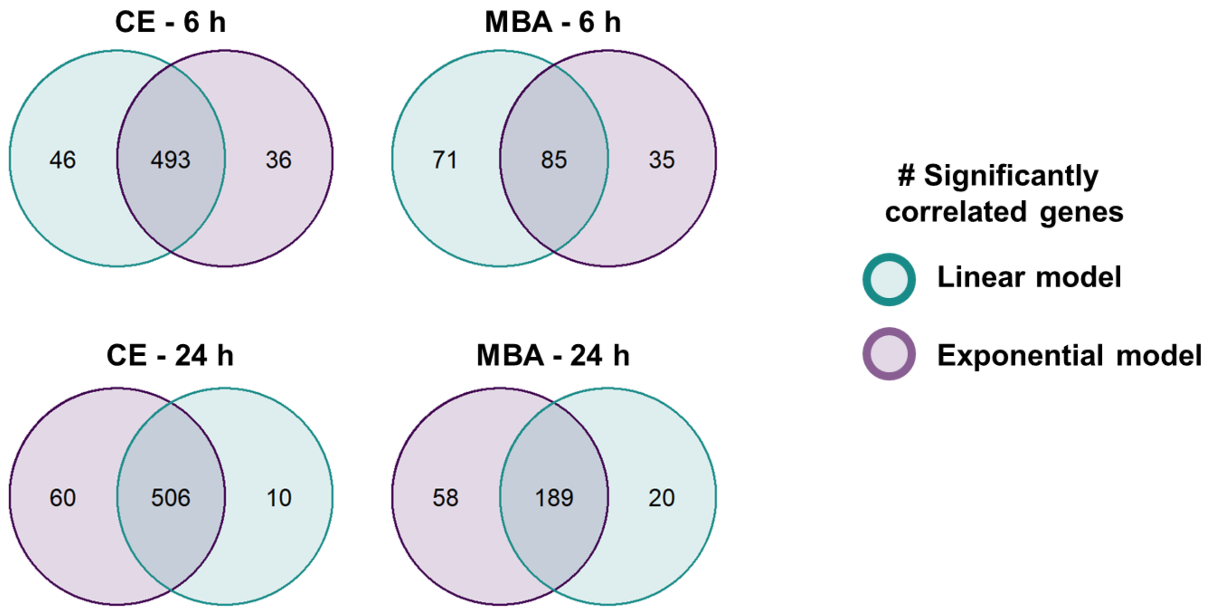

Figure S1: Overlap between correlates identified by pairs of linear and exponential models. A linear model and an exponential model were constructed to identify genes expressed in proportion to CE or MBA at 6 h or 24 h post FUS. Overlaps between significantly (p adjusted < 0.05) correlated genes from each pair of models are shown.

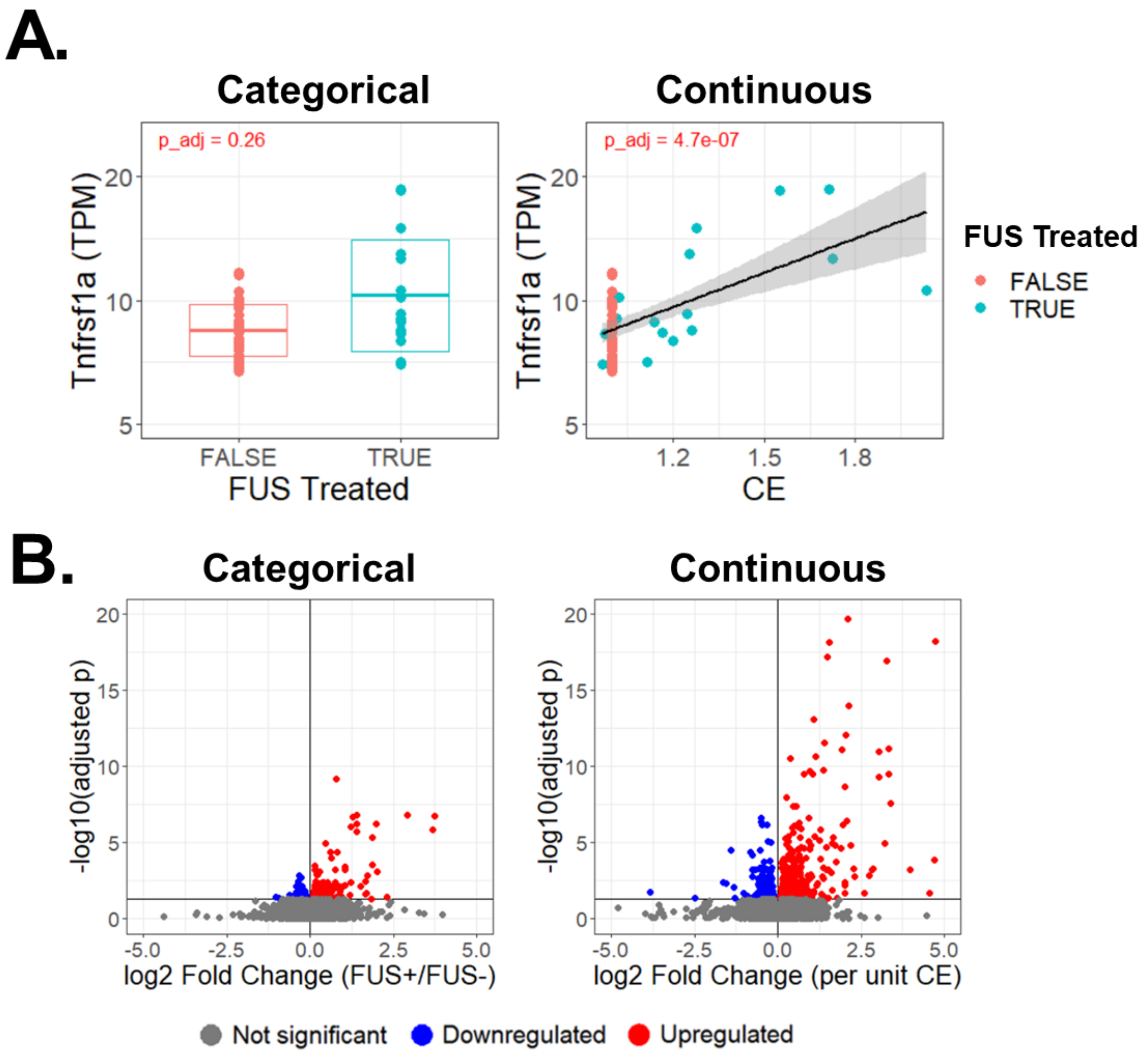

Figure S2: Continuous metrics of BBBD predict gene expression with higher sensitivity than treatment status alone. (A) TPM normalized expression of Tnfrsf1a with data organized according to FUS treated or FUS untreated (left) or according to the extent of CE. Note that the error metrics, namely SEM shown in the categorical plot and the 95% confidence interval in the continuous plot are for visualization purposes only. The adjusted p values on each plot are derived from the Wald test after appropriate negative binomial regression of gene expression, and blockage of the effects of sex, anesthesia, and sequencing batch. (B) Volcano plots of differential gene expression when FUS is treated as a categorical variable (left) or a continuous variable according to CE (right) at 6 h post-treatment. Each dot represents a gene, color coded by the significance of its relative expression (left) or correlation (right).
